# Setting strategy of delay-optimization-oriented SMAC contention window size

**DOI:** 10.1371/journal.pone.0181506

**Published:** 2017-07-21

**Authors:** Yuan Rao, Cheng Deng, Jun Su, Yan Qiao, Jun Zhu, Ru-chuan Wang

**Affiliations:** 1 College of Information and Computer Sciences, Anhui Agricultural University, Hefei, Anhui, China; 2 Jiangsu High Technology Research Key Laboratory for Wireless Sensor Networks, Nanjing, Jiangsu, China; Lanzhou University of Technology, CHINA

## Abstract

Some frame components, such as SYNC (frame synchronization) and RTS/CTS (Ready to Send/Clear to Send), are not taken into consideration when the traditional setting strategies conduct the optimization of SMAC (Sensor MAC) contention window size. This paper proposes mathematical models that allow the analysis of data packets forwarding delay within one SMAC virtual cluster. Simulation results in OMNeT++ show good agreements with the proposed mathematical models, validating the models’ correctness. The curve analyses of the models confirm the existence of delay-optimization-oriented contention window size that is closely related to the number of simultaneously contending nodes. Afterwards, it is shown that SYNC, RTS/CTS and EIFS (Extended InterFrame Space) have impacts on the optimal contention window size and expected delivery delay to various degrees, as well as throughput and energy efficiency. One ideal setting strategy of delay-optimization-oriented SMAC contention window size requires the combination of the network scale, SYNC, RTS/CTS and EIFS. Additionally, it is demonstrated that the proposed setting strategy makes contributions to the improvement in the existing SMAC extensions when they are integrated with each other, in terms of the end-to-end delay, throughput and energy consumption.

## Introduction

As an important underlying network, wireless sensor networks (WSNs) are composed of a large number of sensor nodes deployed in the monitoring areas [1,2]. Sensor nodes deliver data packets to the Sink by constructing multi-hop self-organizing network system. These nodes are usually battery-powered with limited energy capacity. As a result, how to reduce the energy consumption and prolong the network lifetime is of great challenge for WSNs, which great efforts have been made thus far to deal with. However, existing research mainly concentrates on improving network reliability, reducing the hardware cost, optimizing the network protocol, etc [3–6]. As we know, the medium access control (MAC) layer always plays an important foundation for data transmission since it controls the allocation of wireless channel resources. The optimization of MAC protocols is one of important ways for achieving energy savings. As one typical MAC layer protocol for WSNs, SMAC (Sensor MAC) protocol is a modification version of the distributed coordination model in IEEE 802.11 MAC protocol, by introducing sleeping mechanism. In SMAC, the nodes switch their status between active/sleep in order to save energy [7]. It is worth mentioning that a data packet can only be forwarded by one hop in per SMAC cycle. For the purpose of improving SMAC, the adaptive listening mechanism is later introduced into SMAC to increase forwarding distance to 2 hops per operational cycle. However, the improvement is rather limited. As a consequence, the long end-to-end delay makes SMAC inappropriate for applications that require strict timing constraints [8].

In recent years, with the rapid development of sensor technology, SMAC is expected to be improved in order to meet the requirement of many applications, such as, fire monitoring, target tracking, and precision agriculture [9–12]. These applications demand high reliability, less packet loss rate and strict real-time QoS (quality of service) support [13]. To this end, much research attention has been paid to throughput and delay instead of the energy saving. In the past years, comprehensive study on the performance characteristics and disadvantages of SMAC protocol has been conducted to find ways of improving the network performance. It is encouraging that there are great progresses with respect to improving the reliability and timeliness of data transmission [14–18]. More specifically, there have been a variety of extensions of SMAC protocol available, where different network performance parameters are taken into consideration to guarantee different QoS. However, it comes to our attention that almost all of these SMAC extensions, such as T-MAC, RMAC, DMAC and SCP-MAC, focus on improving the multi-hop delay and throughput by trying to notify as many nodes as possible on active routes of incoming data packets as soon as possible [8,19]. Nevertheless, these extensions of SMAC protocol might still suffer from rather poor performance when employed in the bursty and dynamic workload application.

In the network with SMAC-like employed, the channel contention process is an essential procedure when the node initiates data transmission in most occasions except that the channels are already reserved in advance. As a result, the channel contention delay influences the end-to-end delay to a great extent. As we know, the contention window size plays a very important role during the whole contention process [20,21]. Some work has been done to design dynamic contention window mechanism so far. In [22], ASMAC is developed based on the canonical SMAC protocol, and it dynamically adjusts contention window size according to the traffic load in order to achieve the delay reduction and the improvement of the energy efficiency. Unfortunately, the proposed parameterization method is not convincing enough due to the lack of theoretical analysis. The authors in [23] present the analysis about the impact of contention window size on the energy consumption and packet delay under various network scales. They confirm the existence and determination of the optimal contention window size. Whereas, the proposed optimal contention window is still questionable because of not taking into consideration the frame synchronous (SYNC) phase, RTS/CTS (Request to Send/Clear to Send) and EIFS (Extended InterFrame Space) duration. Additionally, the influence of dynamic contention window size on the multi-hop data transmission is not covered yet.

As a remedy, this study is to evaluate the effect of SYNC, RTS/CTS and EIFS on the delay-optimization contention window size for guiding the setting strategy of delay-optimization-oriented contention window size. First of all, the delivery delay within one SMAC virtual cluster is mathematically derived from the perspectives of both single node and the whole network. Second, the correctness of the proposed mathematical models is evaluated by means of simulations. Third, the possible factors that influence the delay-optimization contention window size is thoroughly investigated based on the proposed models for providing one reference for further appropriately adjusting contention window size. Fourth, we evaluate the network performance gain offered by the delay-optimization-oriented dynamic contention window size under two scenarios of one virtual cluster and multi-hop sensor network. Finally, some interesting conclusions are drawn for guiding the setting strategy of delay-optimization-oriented dynamic contention window size.

## SMAC mechanism with adaptive listening mechanism

In SMAC, the frame is divided into two stages: active and sleeping stages, and the duration in the active stage include synchronous and contention periods. In the synchronization period, nodes exchange their schedules by periodically broadcasting a SYNC packet to their immediate neighbors. In the listening period, if a node needs to transmit data, it has to compete with other contending nodes for medium access. When it turns into active stage, the node starts channel listening procedure. After receiving packets, it takes some actions as follows. If the packet contains SYNC information from other nodes, the node sets its own scheduling information according to the SYNC packet. If the packet contains different scheduling information from other nodes, the node would pick up the first arrival packet and record scheduling information. If no scheduling information is received from other nodes, the node generates its own scheduling information and broadcast towards the network. Usually, the virtual cluster is formed by the nodes with the same scheduling, and the boundary nodes within the same cluster need to maintain multiple scheduling information. In order to ensure that the new cluster member node and existing nodes follow the same scheduling information, each node periodically broadcast its own scheduling information at the beginning of the synchronization phase.

**Fig 1:**
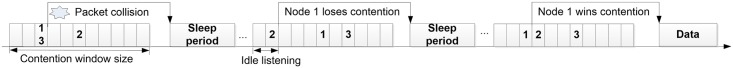
Three possible contention results.

After the synchronous phase, the nodes enter into the contention stage. Nodes randomly select their own time slots from the contention window at the beginning of contention phase. Fig 1 shows the three possible contention results of node slot selection. Assume that there are three contending nodes in the considered network. The following analysis takes Node 1 as an example. Note that Node 1 would go to sleep and remain asleep until the end of transmission once it loses the chance to access channel. 1) Node 1 detects collision and restarts contention after staying asleep for about a period of EIFS (Extended InterFrame Space). 2) Node 1 goes to sleep. In contrast, one of other nodes successfully wins the right of accessing channel, and afterwards starts data transmission. 3) Node 1 wins the contention and starts the data transmission. It is worth noting that the three possible contention results are independent on example node and the total number of nodes.

SMAC uses RTS / CTS mechanism to guarantee the successful transmission. In the case of no collision, the node with the smallest idle listening duration wins the right of accessing channel, then broadcasts RTS packet into the network. The RTS packet contains the time required for the node completing one round of data transmission. Other contending nodes detect RTS packet sent by the winner node and set their sleeping time according to the received NAV(Network Allocation Vector, NAV) value. Moreover, these nodes continue monitoring the channel to detect the collision events by identifying whether CTS packet is received. The sending node starts data transmission after receiving CTS packet. The destination node replies an ACK after receiving the data. If the conflict occurs, all nodes are not able to detect CTS from the destination. Subsequently, the node goes to sleep and remain asleep for a period of EIFS. Afterwards, all nodes join one new round of contention at the end of sleeping.

**Fig 2:**
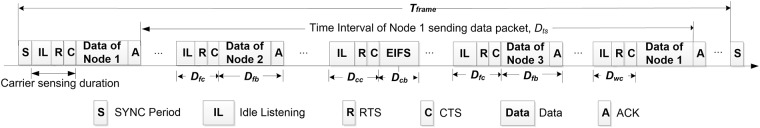
Frame components of SMAC with adaptive listening mechanism.

SMAC adopts self-adaptive listening mechanism to reduce the data delivery delay. The neighbors of active node, instead of remaining asleep until the whole frame duration elapses, immediately joins one new round of contention after the data transmission. The frame structure of SMAC with self-adaptive listening mechanism is illustrated in Fig 2. Taking the two consecutive contentions of Node 1 as example, it describes one typical network contention process. To be more specific, Node 1 suffers from going to sleep several times between its two consecutive contentions since either other nodes win the contention or collision occurs.

Assume that there are *N* contending nodes participating in each round of contention, the contention window size is *W*, the nodes randomly pick up a time slot from the contention window to determine their idle listening duration. Let *T*_*sync*_ represent the duration of SMAC synchronization phase, *T*_*listen*_ denote the sum of the idle listening and RTS/CTS durations. Duty cycle is the ratio of the active duration (*T*_*active*_) and one SMAC frame duration (*T*_*frame*_). Therefore, the SMAC frame length can be determined as follows:

$$\begin{array}{cc} T_{frame} & =T_{active}/\gamma_{dc}=\left(T_{sync}+T_{listen}\right)/\gamma_{dc}\\ & =\left(T_{sync}+(W-1)t_{slot}+t_{RTS}+t_{CTS}\right)/\gamma_{dc} \end{array}$$ (1)

Where *t*_*slot*_ is the single slot duration of contention window. *t*_*RTS*_ and *t*_*CTS*_ represent the transmission duration of the RTS and CTS packet in the channel, respectively.

## Mathematical modeling of the delivery delay

As shown in Fig 2, after completing data transmission, Node 1 is likely to suffer from many collision events or failing in the contention. In these two aforementioned cases, Node 1 has to implement back-off, that is to say, going to sleep and remaining asleep till the beginning of the next round of contention. In this section, Node 1 is selected as an example to investigate the expected time interval between two consecutive successful data transmission for a specific node. The expected time interval, denoted as *D*_*ts*_, is called as the delivery delay derived from perspective of the single node. Afterwards, the delivery delay from the perspective of network is further derived. In order to analyze the effect of each component of SMAC frame on optimal delay contention window, we suppose that the queue of the nodes is always full, and all nodes immediately participate in the next round of contention after the completion of data transmission. Table 1 lists of some important notations and their meanings to be used in our analysis.

**Table 1:** List of notations and variables.

Symbol	Description	Symbol	Description
*D*_*wc*_	Expected idle listening duration in the case of winning the contentions*	*D*_*cb*_	Expected sleeping duration in the case of collision occurrence*
*D*_*fc*_	Expected idle listening duration in the case of failing in the contentions*	*D*_*bo*_	Expected sleeping duration in the case of failing in the contentions*
*D*_*cc*_	Expected idle listening duration in the case of collision occurrence*	*D*_*ts*_	Expected time interval between two consecutive medium access events*
*D*_*con*_	Expected idle listening duration between two consecutive data transmissions*	*D*_*net*_	Expected time interval between two consecutive medium access events from network view*
*D*_*fb*_	Expected sleeping duration in the case of contention failure*	*β*	Expected collision times between successive non-collisions
*V*	Expected data transmission times within one SMAC frame	*μ*	Expected collision times between successive medium release and access
*W*	Data transmission CW size	*φ*	First occupied slot Index during one contention
*p*_*nc*_	Non-collision probability during one contention	*T*_*listen*_	Duration of listening period
*p*_*c*_	Collision probability during one contention	*T*_*active*_	Duration of active period
*N*	Number of contending nodes	*t*_*slot*_	Duration of single contention slot
*m*	Number of collision nodes	*t*_*EIFS*_	Duration of an Extended Interframe Space
*ψ*	Variable representing the first occupied slot	*t*_*DATA*_	Duration of single DATA packet
ϒ	Contention result during one contention	*t*_*RTS*_	Duration of single RTS packet
*T*_*frame*_	Duration of a SMAC frame	*t*_*CTS*_	Duration of single CTS packet
*T*_*sync*_	Duration of a synchronization period	*t*_*ACK*_	Duration of single ACK packet

* These values are evaluated from the perspective of any given node.

### Expected idle listening duration

Firstly, we focus on the mathematical modeling of the expected idle listening delay that any given node (say **Node 1** in the Fig 2) takes to get the right of accessing medium, *D*_*wc*_, during one round of contention. The derivation of *D*_*wc*_ is based on the expected probability that the **Node 1** succeeds in winning one round of contention. Let P[*ψ* = *φ*, ϒ = *success*] represent the probability that the first occupied slot is only selected by the **Node 1**. There are *W*^*N*^ different slot assignments among which the following assignment results in non-collision transmission: *φ* is chosen by the **Node 1** and the slots *φ* + 1 to *W*, i.e., *W* − *φ* slots, are chosen randomly by other *N* − 1 nodes. As a result,

$$P\left[\psi =\varphi ,ϒ=success\right]=\frac{{\left(W-\varphi \right)}^{N-1}}{W^{N}}$$ (2)

And the expected probability of **Node 1** winning the contention can be calculated as follows,

$$P\left[ϒ=success\right]=\sum_{\varphi =1}^{W-1}P\left[\psi =\varphi ,ϒ=success\right]=\sum_{\varphi \text{=}1}^{W-1}\frac{{\left(W-\varphi \right)}^{N-1}}{W^{N}}$$ (3)

Hence, the expected idle listening duration that the **Node 1** takes to win one round of contention, *D*_*wc*_, is

$$D_{wc}=\sum_{\varphi =1}^{W-1}\left(P\left[\psi =\varphi |ϒ=success\right]\left(\left(\varphi -1\right)t_{slot}+t_{RTS}+t_{CTS}\right)\right)$$ (4)

Where by the definition of the conditional probability,

$$P\left[\psi =\varphi |ϒ=success\right]=\frac{P\left[\psi =\varphi ,ϒ=success\right]}{P\left[ϒ=success\right]}=\frac{{\left(W-\varphi \right)}^{N-1}}{\sum_{\varphi =1}^{W}{\left(W-\varphi \right)}^{N-1}}$$ (5)

Similarly, the expected idle listening duration that **Node 1** has to suffer from the contention failure, *D*_*fc*_, is

$$D_{fc}=\left(N-1\right)\sum_{\varphi =1}^{W-1}\left(P\left[\psi =\varphi |ϒ=lose\right]\left(\left(\varphi -1\right)t_{slot}+t_{RTS}+t_{CTS}\right)\right)$$ (6)

Where P[*ψ* = *φ*| ϒ = *lose*] represents the probability that the first occupied slot index, *φ*, is selected by other nodes and there is no collision occurrence, and it can be derived in the same way as P[*ψ* = *φ*, ϒ = *success*].

Next, we focus on the case of collision occurrence during the contention process. Since each contention is independent and random, the contention can be modeled as a Bernoulli trial with non-collision and collision probabilities of *p*_*nc*_ and *p*_*c*_ [23]. The expected times of collision occurrence between two consecutive non-collision events during the continuous contentions, *β* are found to be,

$$\beta =\frac{1}{p_{nc}}-1$$ (7)

Where, the probability of non-collision occurrence, *p*_*nc*_, is calculated as,

$$p_{nc}=\sum_{\varphi =1}^{W-1}\left(P\left[\psi =\varphi ,ϒ=success\right]+P\left[\psi =\varphi ,ϒ=lose\right]\right)=N\frac{\sum_{\varphi \text{=}1}^{W-1}{\left(W-\varphi \right)}^{N-1}}{W^{N}}$$ (8)

If there is no collision occurrence in the network with *N* contention nodes, the contention times are *N* between two consecutive channel access events of any specific node. Hence, the expected times of collision occurrence between two consecutive channel access events are calculated as *μ* = *Nβ*. The expected idle listening duration that **Node 1** suffers from the collision contention is

$$D_{cc}=\mu \sum_{\varphi =1}^{W-1}\left(P\left[\psi =\varphi |ϒ=collision\right]\left(\left(\varphi -1\right){\text{t}}_{slot}+t_{RTS}+t_{CTS}\right)\right)$$ (9)

Where

P[ψ=φ|ϒ=collision]=P[ψ=φ,ϒ=collision]1−pnc (10)

P[*ψ* = *φ*| ϒ = *collision*] represents the probability in which the first occupied slot is *φ* in the case of collision occurrence. Assume that there are *m* nodes that select *φ* as their occupied slot, the remained *W*–*φ* slots, between *φ*+1 to *W*, are chosen by *N-m* nodes. Hence,

$$P\left[\psi =\varphi ,ϒ=collision\right]=\frac{\sum_{m=2}^{N}\left({}_{m}^{N}\right){\left(W-\varphi \right)}^{N-m}}{W^{N}}$$ (11)

Finally, the expected idle listening duration between two consecutive data transmissions can be calculated as the sum of the three components mentioned above.

$$\begin{array}{ll} D_{con} & =D_{wc}+D_{fc}+D_{cc}\\ & =\sum_{\varphi =1}^{W-1}\left(P\left[\psi =\varphi |ϒ=success\right]\left(\left(\varphi -1\right){\text{t}}_{slot}+t_{RTS}+t_{CTS}\right)\right)\\ & \;+\left(N-1\right)\sum_{\varphi =1}^{W-1}\left(P\left[\psi =\varphi |ϒ=lose\right]\left(\left(\varphi -1\right){\text{t}}_{slot}+t_{RTS}+t_{CTS}\right)\right)\\ & \;+\mu \sum_{\varphi =1}^{W-1}\left(P\left[\psi =\varphi |ϒ=collision\right]\left(\left(\varphi -1\right){\text{t}}_{slot}+t_{RTS}+t_{CTS}\right)\right) \end{array}$$ (12)

### Expected back-off duration

Node has to go to sleep if there is a collision occurrence or it fails in winning the contention. Similar to the derivation of *D*_*fc*_ and *D*_*cc*_, it is easy to obtain the expected sleeping duration that **Node 1** spends after its contention failure,

$$D_{fb}=\left(N-1\right)\sum_{\varphi =1}^{W-1}P\left[\psi =\varphi |ϒ=lose\right]\left(t_{DATA}+t_{ACK}\right)$$ (13)

Similarly, the expected sleeping duration that **Node 1** spends after the collision occurrence is,

$$D_{cb}=\mu \left(\sum_{\varphi =1}^{W-1}P\left[\psi =\varphi |ϒ=collision\right]t_{EIFS}\right)$$ (14)

Then, the expected sleeping duration that **Node 1** spends between two consecutive data transmissions,

$$\begin{array}{ll} D_{bo} & =D_{fb}+D_{cb}\\ & =\left(N-1\right)\sum_{\varphi =1}^{W-1}\text{P}\left[\psi =\varphi |ϒ=lose\right]\left(t_{DATA}+t_{ACK}\right)+\;\mu \left(\sum_{\varphi =1}^{W-1}\text{P}\left[\psi =\varphi |ϒ=collision\right]t_{EIFS}\right) \end{array}$$ (15)

### Expected delivery delay of data packet

The times of the successful data transmission within one entire SMAC frame (*V*) is calculated as the difference of *T*_*frame*_ and *T*_*sync*_ divided by the sum of the expected data transmission and collision durations. Hence, the value of *V* is calculated as follows,

$$V=\frac{T_{frame}{\text{-T}}_{sync}}{\left(\left(\left(\frac{W}{N+1}\right)t_{slot}+t_{RTS}+t_{CTS}{\text{+t}}_{DATA}+t_{ACK}\right)+\beta \left(\left(\frac{W}{N+1}\right)t_{slot}+t_{RTS}+t_{CTS}{\text{+t}}_{EIFS}\right)\right)}$$ (16)

The delivery delay for **Node 1** is the sum of its idle listening duration, sleeping duration, synchronization duration, and data transmission duration. As a result, the expected delivery delay of **Node 1** is equal to,

$$\begin{array}{ll} D_{\text{ts}} & =\left(\frac{N}{V}\right)T_{sync}+\sum_{\varphi =1}^{W-1}P\left[\psi =\varphi |ϒ=success\right]\left(\left(\varphi -1\right)t_{slot}+t_{RTS}+t_{CTS}+t_{DATA}\right)\\ & \;+\left(N-1\right)\sum_{\varphi =1}^{W-1}P\left[\psi =\varphi |ϒ=lose\right]\left(\left(\varphi -1\right)t_{slot}+t_{RTS}+t_{CTS}+t_{DATA}+t_{ACK}\right)\\ & \;+\;\mu \left(P\left[\psi =\varphi |ϒ=collision\right]\left(\left(\varphi -1\right)t_{slot}+t_{RTS}+t_{CTS}+t_{EIFS}\right)\right) \end{array}$$ (17)

Different from the delivery delay derived from the perspective of single node, the delivery delay derived from the perspective of the whole network does not take into consideration the delay from the contention failure. Combining the Eqs (6), (7) and (9), it can be found that the delivery from the perspective of the whole network is,

$$\begin{array}{ll} D_{\text{net}} & =\frac{T_{sync}}{V}+\sum_{\varphi =1}^{W-1}P\left[\psi =\varphi |ϒ=success\right]\left(\left(\varphi -1\right){\text{t}}_{slot}+t_{RTS}+t_{CTS}+t_{DATA}\right)\\ & +\beta \;\left(\sum_{\varphi =1}^{W-1}P\left[\psi =\varphi |ϒ=collision\right]\left(\left(\varphi -1\right){\text{t}}_{slot}+t_{RTS}+t_{CTS}+t_{EIFS}\right)\right) \end{array}$$ (18)

In conclusion, the two formulas above are exactly the mathematical models of the delivery delay from the perspectives of single node and the whole network, respectively. According to the derived models, the main factors that influence the delivery delay involve the number of contending nodes, the contention window size, SYNC duration, RTS/CTS duration, EIFS, the durations of data transmission and ACK. Such phenomenon indicates that the SMAC frame components, including SYNC, RTS/CTS and EIFS, have impacts on the delivery delay as well as contention window size. However, as shown in formulas (17) and (18), it is difficult to quantify the relationships among the delivery delay, contention window size and SMAC frame components by performing the mathematical analysis due to the models’ complexity.

**Fig 3:**
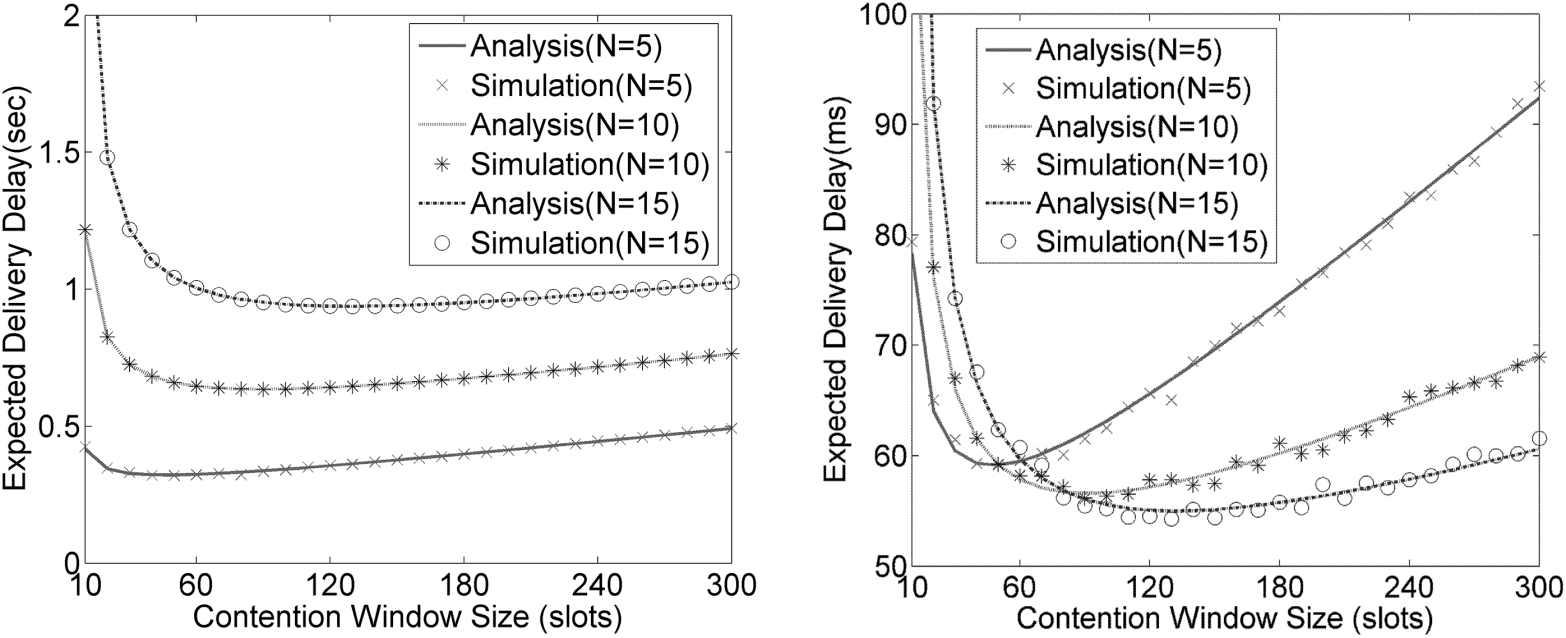
The delivery delay of data packet (Node and Network perspectives). 3a. The delivery delay, *Dts* (Node perspective). 3b. The delivery delay, *Dnet* (Network perspective).

Particularly, as shown in Fig 3 below, each curve of expected delivery delay, when regarding the contention window size as only independent variable, has only one minimum point. Therefore, based on the theory of advanced mathematics, it is theoretically feasible to quantify the delay optimization by performing mathematical analysis of the formulas (17) and (18). The procedure goes as follows. We take the partial derivative of the formulas (17) and (18) with respect to the number of contention window size. Afterwards, by setting the right of the derived formulas equal to zero, and solving equations, the optimal contention window size could be obtained, and it is determined by other variables, such as, the number of contending nodes, SYNC and RTS/CTS durations. Finally, we get the expression of the optimized delivery delay by plugging the optimal contention window size into the formulas (17) and (18). However, it is found out that the derived equations about the optimal contention window size are hard to solve due to the equation complexity. In addition, even if the equations were solved, the equations of the optimized delivery delay would be too complicated to explicitly demonstrate the optimization results.

Therefore, similar in [20, 23], in the following section, the combination of simulations and curve analyses is implemented to explore the interrelationships among the delivery delay, contention window size and SMAC frame components. More specifically, the verification of the mathematical delay models in Eqs (17) and (18) is implemented by means of simulation conductions. Afterwards, based on the curve analyses of the models, we investigate the existence of the delay-optimization-oriented contention window size, and further evaluate the influence of the aforementioned SMAC frame components on the delay-optimization-oriented contention window size and expected delivery delay. Finally, through simulations, the throughput and energy consumption are inspected considering the dynamic contending nodes along with corresponding optimal contention window size. Additionally, we thoroughly evaluate the network performance gains offered by the dynamic contention window strategy over the existing SMAC extensions.

## Results and discussion

Two groups of investigations are conducted in order to verify the mathematical delay models and the existence of the delay-optimization-oriented contention window size, as well as thoroughly to evaluate the influence of dynamic contention window strategy on network performance within one virtual cluster and multi-hop sensor network. Subsequently, the discussion made to conclude the setting strategy of delay-optimization-oriented contention window size.

The first group focuses on the scenario within one virtual cluster. For the purpose of verifying the correctness of the mathematical delay models in previous section, several simulations are carried out in OMNeT++[24], in an square area of 30 m by 30 m. The Sink is located at the center of area. All nodes set the Sink as their only destination. The events are logged from the start of simulation till its end. The simulations are run for 30 times, and the duration of each simulation is 1000 seconds. The main simulation parameters are listed in Table 2. Afterwards, using MATLAB, the curve analyses of the models are implemented to investigate the existence of the optimal contention window size, and to evaluate the influence of the SMAC frame components on the delay-optimization-oriented contention window size and expected delivery delay. Last but not the least, through simulations, the influence of SMAC frame components on the throughput and energy consumption are explored considering the dynamic contending nodes along with corresponding optimal contention window size.

**Table 2:** Simulation parameters.

Parameter	Value
**Contending nodes**	2–20 nodes
**Duty cycle**	40%
**Channel bitrate**	250 Kb/s
**Data packet size**	1000 bits
**Data contention window size**	10–300 slots (ms)
**SYNC duration**	31 slots (ms)
**EIFS duration**	30 ms
**RTS/CTS/ACK duration**	9 ms
**Communication range**	40 m

The other group is devoted to comparing the dynamic contention window strategy against the existing SMAC extensions. More specifically, the simulations are implemented in order to evaluate the network performance gains offered by the dynamic contention window strategy over the SMAC extensions designed for the multi-hop data transmission. During our simulations, the parameters in Table 2 are employed except the number of contending nodes. In our case, the realistic 200-node network in [19] is used, where 200 sensor nodes are uniform randomly deployed in a square area of 2000 m by 2000 m, the Sink is located at the center of the square. The maximum path length from a sensor to the Sink is 15 hops. In comparison with the pretty light traffic in [19], more realistic traffic model is employed. Traffic loads are generated by constant bit rate (CBR) flows. 140 of 200 nodes generate one data packet every 50 seconds. The rest nodes generate data every 10 seconds to mimic the bursty traffic. As a consequence, a light traffic load is injected into the target sensor network. Each performance metric is averaged from the results of 30 random runs. Note that the same energy consumption models of sensor node in [19] are employed in the two groups of investigations above.

### Model verification and optimal contention window size

The number of contending nodes and contention window size are taken as variables to verify the delay models and to confirm the existence of the optimal contention window size. Fig 3 shows the analysis and simulation results about the data delivery delay from the perspectives of single node and the whole network. As can be seen in Fig 3, the results of analyses and simulations highly match with each other. The difference between the two aforementioned delivery delays can be explained that the delivery delay derived from the perspective of the whole network does not take into account the delay caused by the contention failure.

It is worthwhile to mention that there are similar changing trends between the delivery delays from the perspectives of single node and the network. Apparently, both of them tend to rise steadily after a sharp decline with the increase of contention window size. As a matter of fact, the data delivery delay is affected by both contention window size and collision probability for any fixed network scales. In addition, the change trends of both contention window size and collision probability are opposite. When the contention window size is small, the collision probability is relatively large, which can be reduced by increasing the contention window size. With the increase of the contention window size, the collision probability becomes less and less whereas the idle listening duration would increase. Before the delivery delay reaches the minimum, the collision probability starts to play a dominant role in the expected delivery delay. Afterwards, the idle listening duration takes control of the change in the delivery delay. The optimal compromise between idle listening duration and collision probability exactly corresponds to the minimum delivery delay in Fig 3.

**Fig 4:**
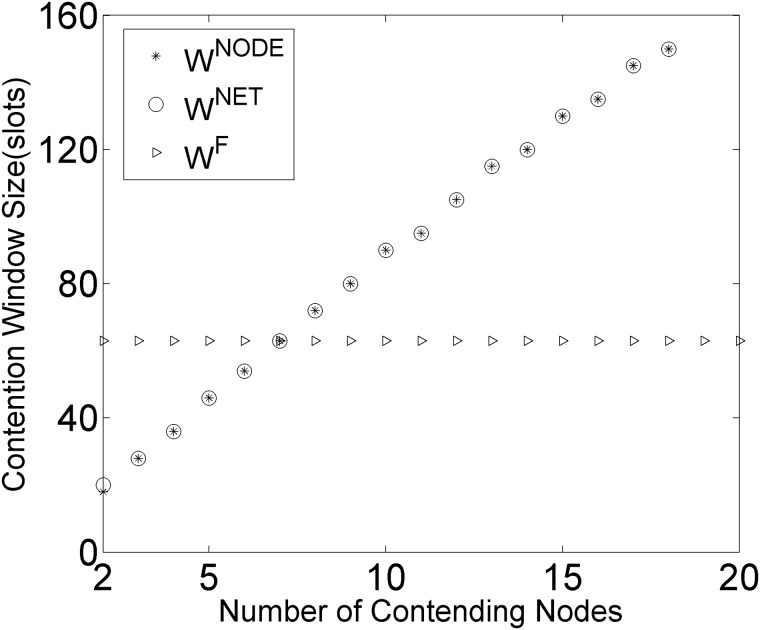
The optimal contention window size(Perspectives of Node and Network).

Fig 4 illustrates the optimal delay contention window size corresponding to the minimum delay points under the condition of various contending nodes, as well as traditional fixed contention window size in SMAC. W^NODE^, W^NET^ are optimal contention window sizes obtained from the perspective of single node and the whole network, respectively. W^F^ represents the fixed contention window size of 63 offered by the canonical SMAC. Both W^NODE^ and W^NET^ tend to rise rapidly with the increase of contending node, which is significantly different from the traditional SMAC. According to Eqs (17) and (18), *D*_*ts*_ is *N* times of *D*_*net*_, therefore, the lowest points are approximate with regards to two curves of delivery delay for the same number of contending nodes, which matches the results presented in Fig 4. Apparently, the correctness of the delivery delay models is verified.

As depicted in Fig 3, the change trend of *D*_*net*_ is much sharper compared with *D*_*ts*_. This should be ascribed to the differences in y-axis interval granularity and in the ranges of *D*_*ts*_ and *D*_*net*_. To better illustrate the analytical results, *D*_*net*_ is employed to evaluate the effect of SMAC frame components on the optimal contention window size, expected delivery delay, throughput and energy efficiency in the following section.

### Influence of SMAC frame components

In this section, we focus on demonstrating the effect of the optimal contention window size and SMAC frame components on the three key performance metrics: delivery delay, energy consumption and throughput. To make our description clear, **E**, **R** and **S** are used to represent EIFS, RTS/CTS and SYNC durations. As tabulated in Table 3, W^E^, W^SE^, W^RE^ and W^SRE^ represent the cases of EIFS only considered, EIFS and SYNC durations considered, RTS/CTS and EIFS considered, RTS/CTS and SYNC durations considered, all three components considered.

**Table 3:** Combinations of SMAC frame components.

Parameter	Three Components		
	SYNC	RTS/CTS	EIFS
**W**^**E**^	╳	╳	√
**W**^**SE**^	√	╳	√
**W**^**RE**^	╳	√	√
**W**^**SR**^	√	√	╳
**W**^**SRE**^	√	√	√

√ component considered,

╳ component not considered.

**Fig 5:**
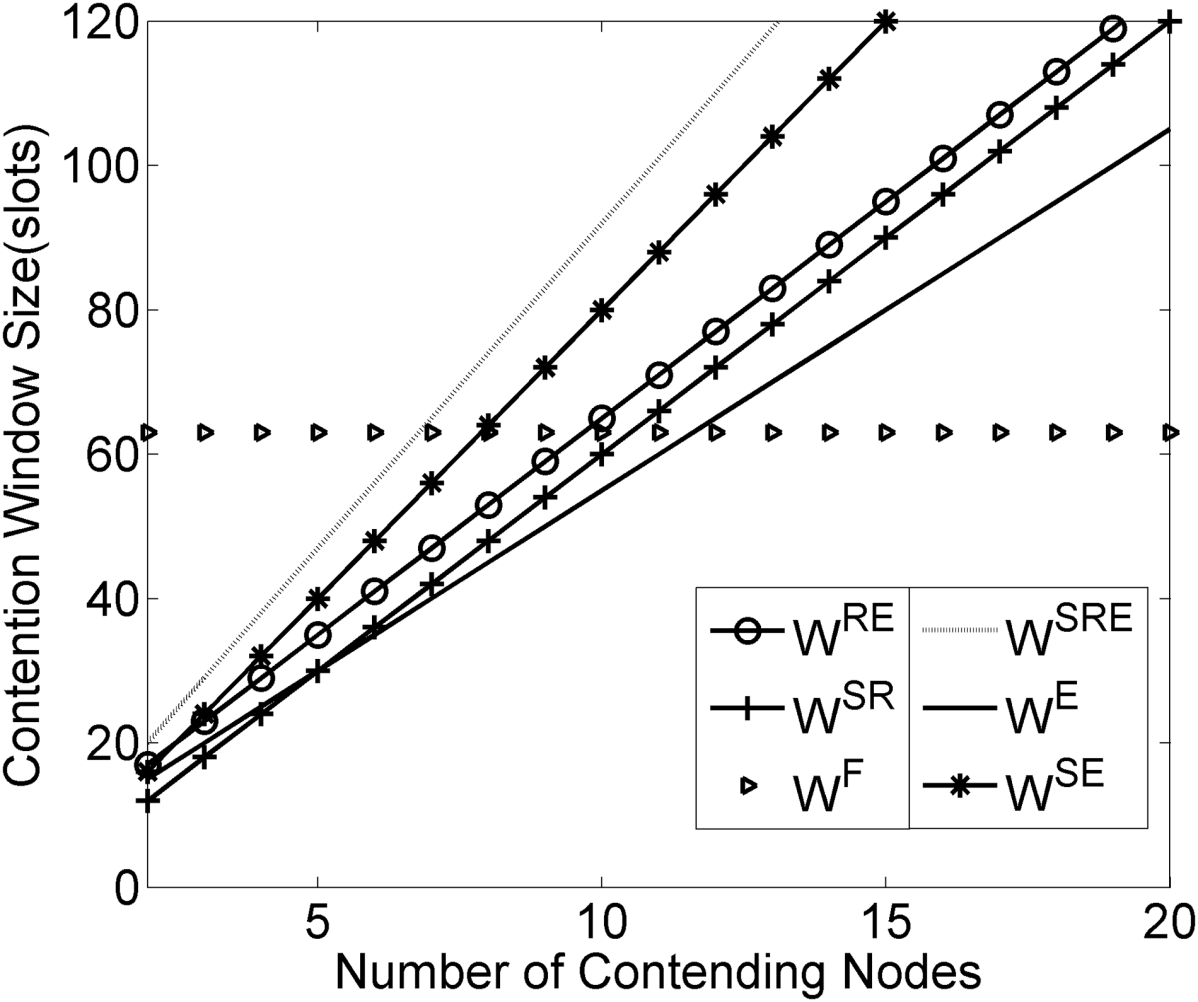
Influence of frame components on optimal CW size.

First of all, we focus on the change characteristics in *D*_*net*_ in the case of the different combinations of the three components to evaluate the effect of SYNC duration, RTS/CTS and EIFS on optimal contention window size. Fig 5 presents the effect of frame components on optimal window size under six combinations of three SMAC frame components. With the increase of contending nodes, the contention window size keeps increasing except W^F^. It should be emphasized that W^SRE^ has the greatest rising rate, and the rest follow the declining order, W^SE^> W^RE^>W^SR^>W^E^, which demonstrates that three components of SMAC frame have influence on the optimal contention window size. Particularly, the impact significance of RTS/CTS, SYNC and EIFS sequentially increases.

Our next objective is to show the influence of the optimal contention window size and SMAC frame components on the following three critical performance metrics for wireless sensor networks: expected delivery delay, throughput and energy consumption. It should be pointed out that the energy consumption is from the average amount of energy that all nodes consume within the same virtual cluster after completing each round of simulation.

**Fig 6:**
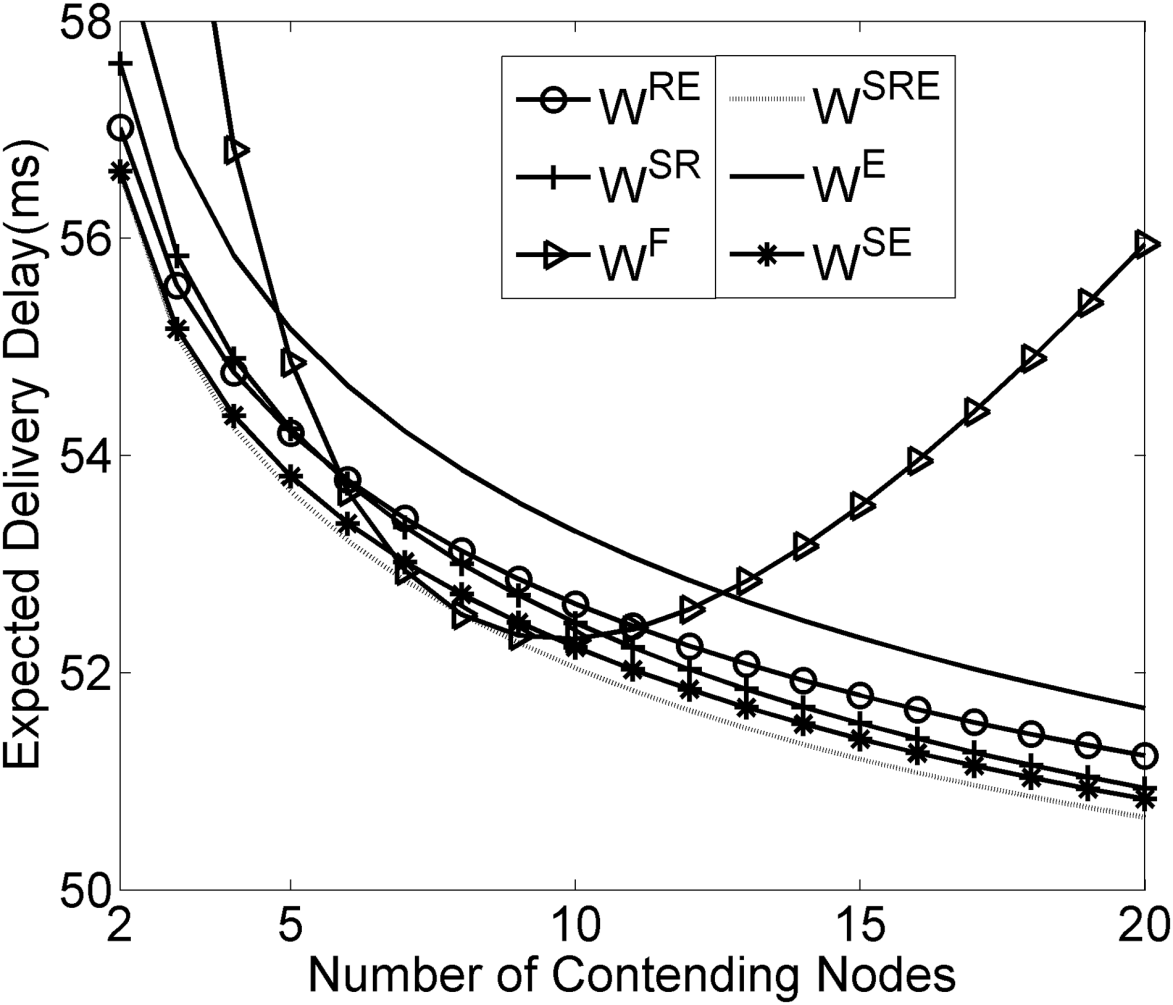
Influence of frame components on delivery delay.

**Fig 7:**
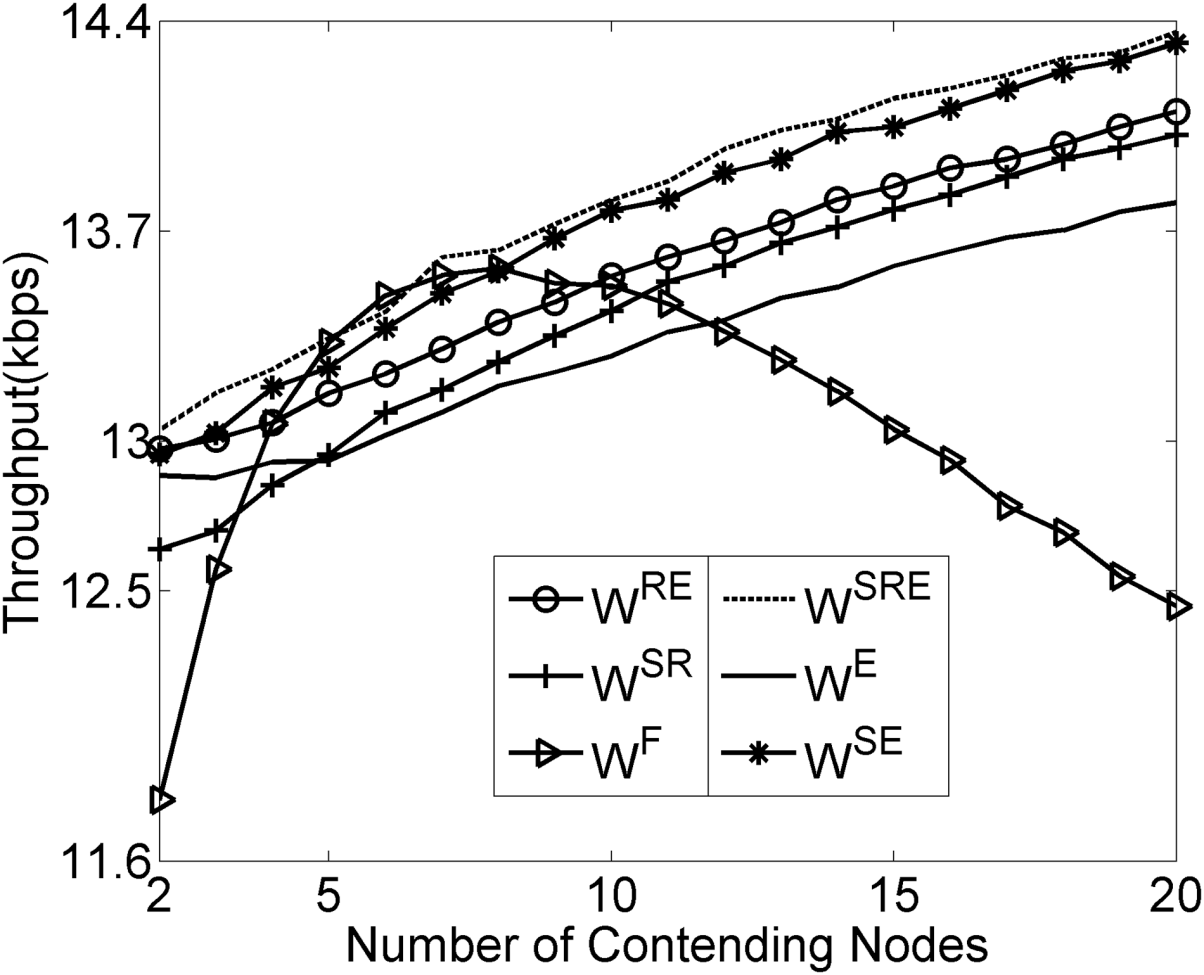
Influence of frame components on throughput.

**Fig 8:**
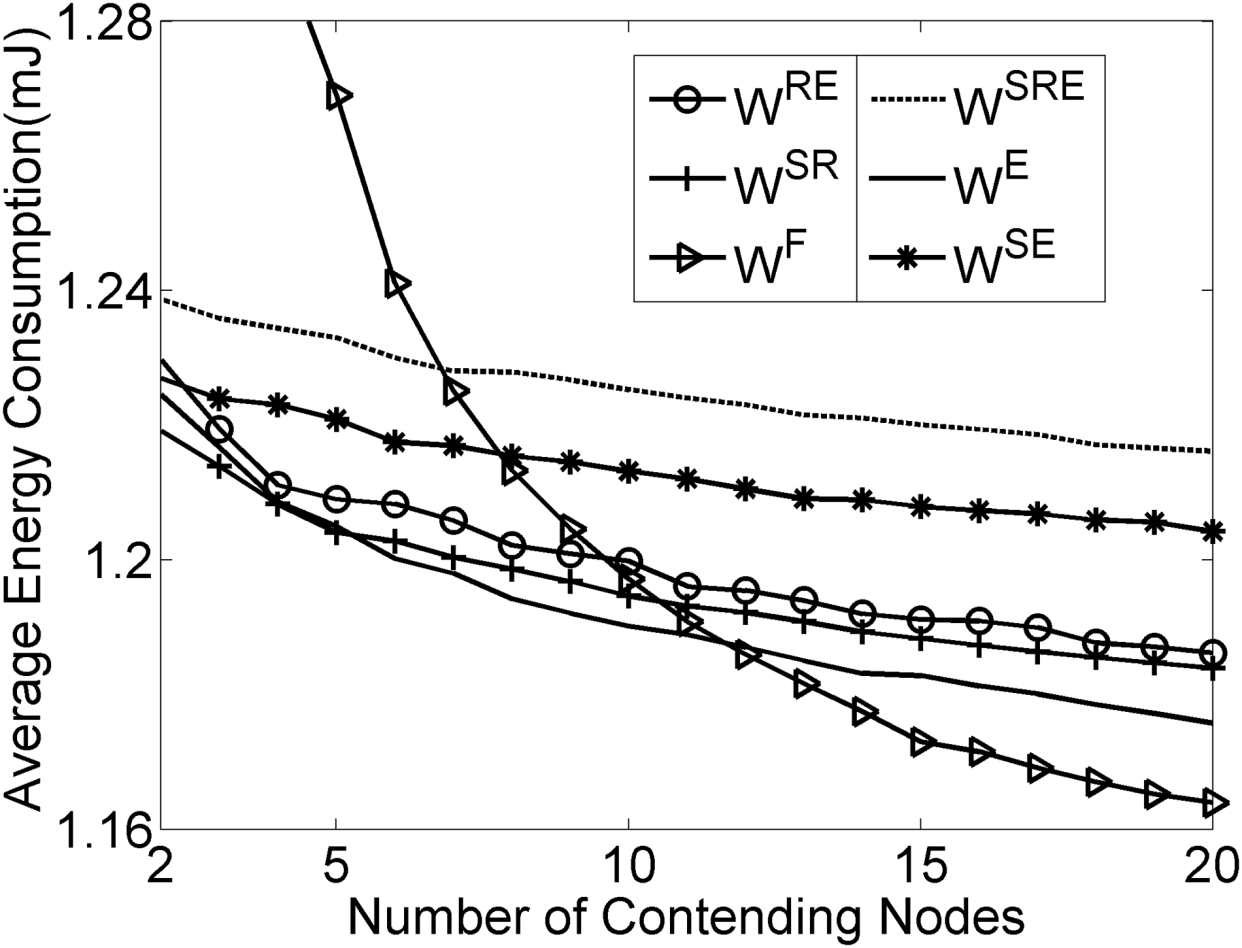
Influence of frame components on energy consumption.

As depicted in Figs 6–8, the results reveal that the three considered performance metrics are closely associated with the densities of contending nodes, which directly demonstrates that the canonical SMAC has drawbacks in dealing with the medium contention under the condition of various node densities. Compared with the fixed contention window size, the dynamic contention window strategy has imposed obviously significant optimization in the expected delivery delay, throughput and energy consumption, especially under the condition of high node densities. Additionally, Figs 6–8 show that among the SMAC frame components, RTS/CTS, SYNC and EIFS sequentially follow an increasing order with respect to the degree of their impacting the expected delivery delay, throughput and energy consumption, which is consistent with the impact significance of RTS/CTS, SYNC and EIFS on the contention window size.

More specifically, it is interesting, from Fig 6, to observe that the curve of expected delivery delay behaves nearly like an upward parabola. This should be ascribed to the fact that the canonical SMAC suffers the less collision events and greater idle listening durations when there are fewer contending nodes. As the contending nodes increase, the collision events tend to occur more and more frequently, meanwhile, the idle listening durations tend to become shorter and shorter. The delivery delay exhibits a continuous decrease until the lowest point, afterwards, turns into an increasing trend as a result of the mutual interaction of the collision occurrence and idle listening duration. It should be noticed that Fig 7 shows that the curve of expected throughput behaves nearly like a downward parabola, which is exactly opposite to the delivery delay in Fig 6. It is definitely reasonable that one greater delay leads to the less amount of throughput.

It should be emphasized that the curves in Figs 6 and 7 clearly show that the dynamic contention window strategy does help in achieving an improvement in the expected delivery delay and throughput. The two figures strongly demonstrate that it is beneficial to integrate the dynamic contention window strategy, instead of the fixed contention window size, with the canonical SMAC. The dynamic contention window strategy reduces idle listening duration by automatically narrowing the contention window size to when there are fewer contending nodes. In the meantime, the increase in contention window size effectively reduces the delay caused by the collision events. It is discovered that the dynamic strategy obtains far less collision probability than the canonical SMAC in the case of high node densities. As a result, the dynamic strategy achieves one relatively steady tradeoff between the collision probability and idle listening duration resulting from the simultaneously appropriate increase in the contention window size with the contending nodes.

As can be seen in Fig 8, the dynamic contention window strategy continuously brings gains in the energy efficiency with the increase in the densities of contending nodes. Intuitively, it is witnessed from Fig 8 that the dynamic strategy consumes more energy than the canonical SMAC in the case of high node densities. This can be explained by investigating the determining factors of the energy consumption. As we know, the energy consumption is positively proportional to the idle listening duration in addition to the amount of transmission data. In our case, the dynamic strategy brings the increase in throughput as well as in energy consumption. As shown in Fig 5, the dynamic contention window strategy uses greater contention window size than the canonical SMAC under the condition of high node densities, which causes the increase in idle listening duration, consequently contributes to the increase in the energy consumption. Thus, it can reasonably be concluded that the improvement in the delivery delay and throughput over the canonical SMAC is achieved at the expense of more energy consumption. Nevertheless, it should be pointed out that the dynamic strategy offers higher energy utilization rate (calculated as energy consumption divided by throughput) compared with the fixed window size.

### Comparisons with existing SMAC extensions

It should be noticed that most of the existing SMAC extensions, such as T-MAC and RMAC, aims at improving the multi-hop data transmission delay and energy consumption by trying to notify as many nodes as possible on active routes of incoming data packets as soon as possible. Most of them follow the fixed contention window size in the canonical SMAC, instead of taking into consideration the influence of contention window size on the delivery delay. Consequently, the existing SMAC extensions follow almost the same performance as the canonical SMAC, which is presented as W^F^ in Figs 4–8 during the channel contention within one virtual cluster. Some existing SMAC extensions are claimed to improve the multi-hop data transmission for the scenarios with temporary bursty traffic. As a matter of fact, in such cases, the contending nodes and traffic load are inevitably dynamic. From this perspective, the dynamic contention window strategy provides one useful complement to the existing SMAC extensions. In our case, RMAC (Routing enhanced MAC protocol) [19] is chosen since it is one typical SMAC extension designed for efficient multi-hop transmission of the bursty traffic. To simplify our evaluations, we assume that there is a routing protocol deployed to provide the shortest path between any two nodes [19]. The node immediately participates in the next round of contention after the completion of data transmission only if there are packets in the queues. Note that the realistic traffic model is employed during our simulation in comparison with the pretty light traffic in [19].

**Fig 9:**
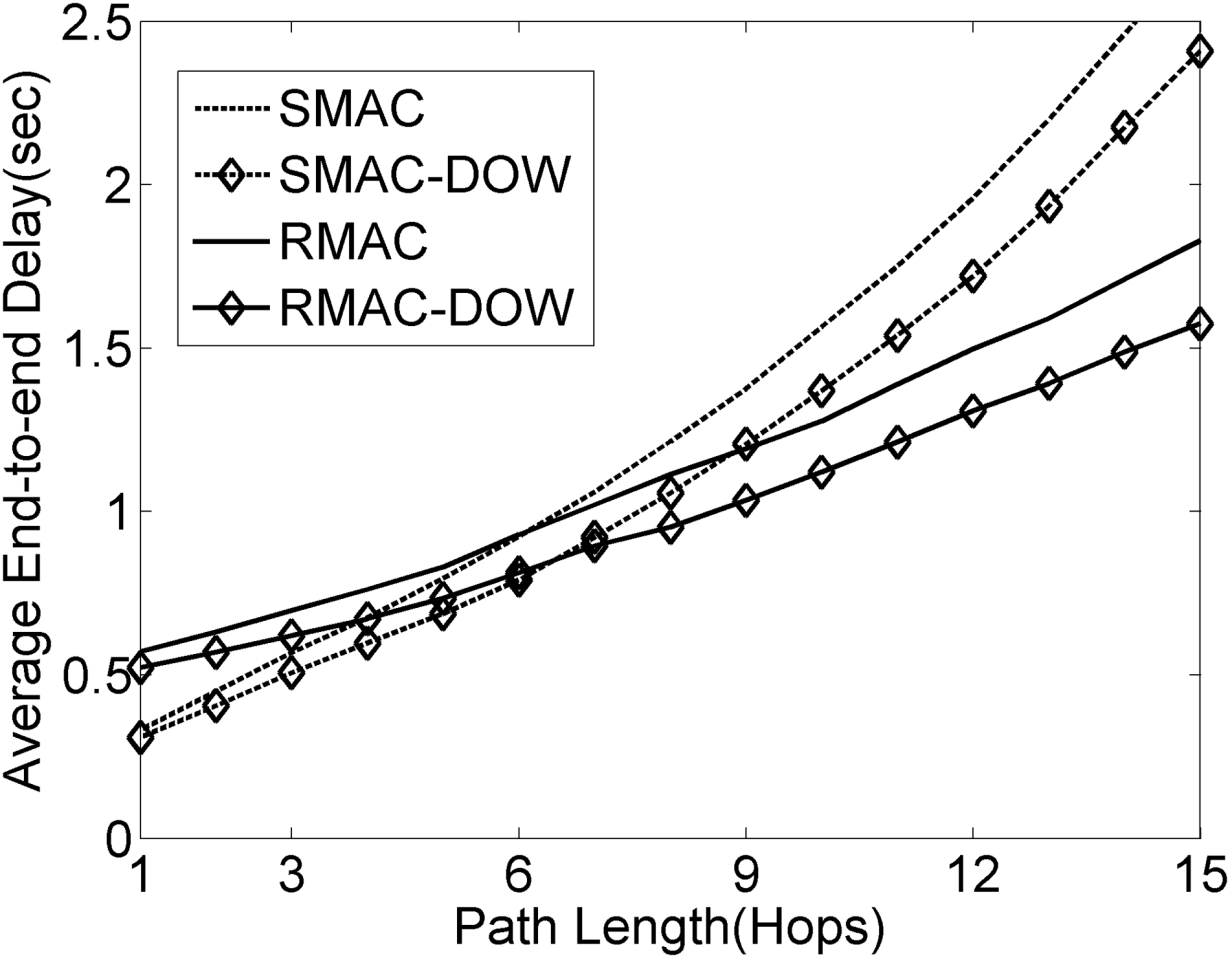
Average end-to-end delay versus hop count.

**Fig 10:**
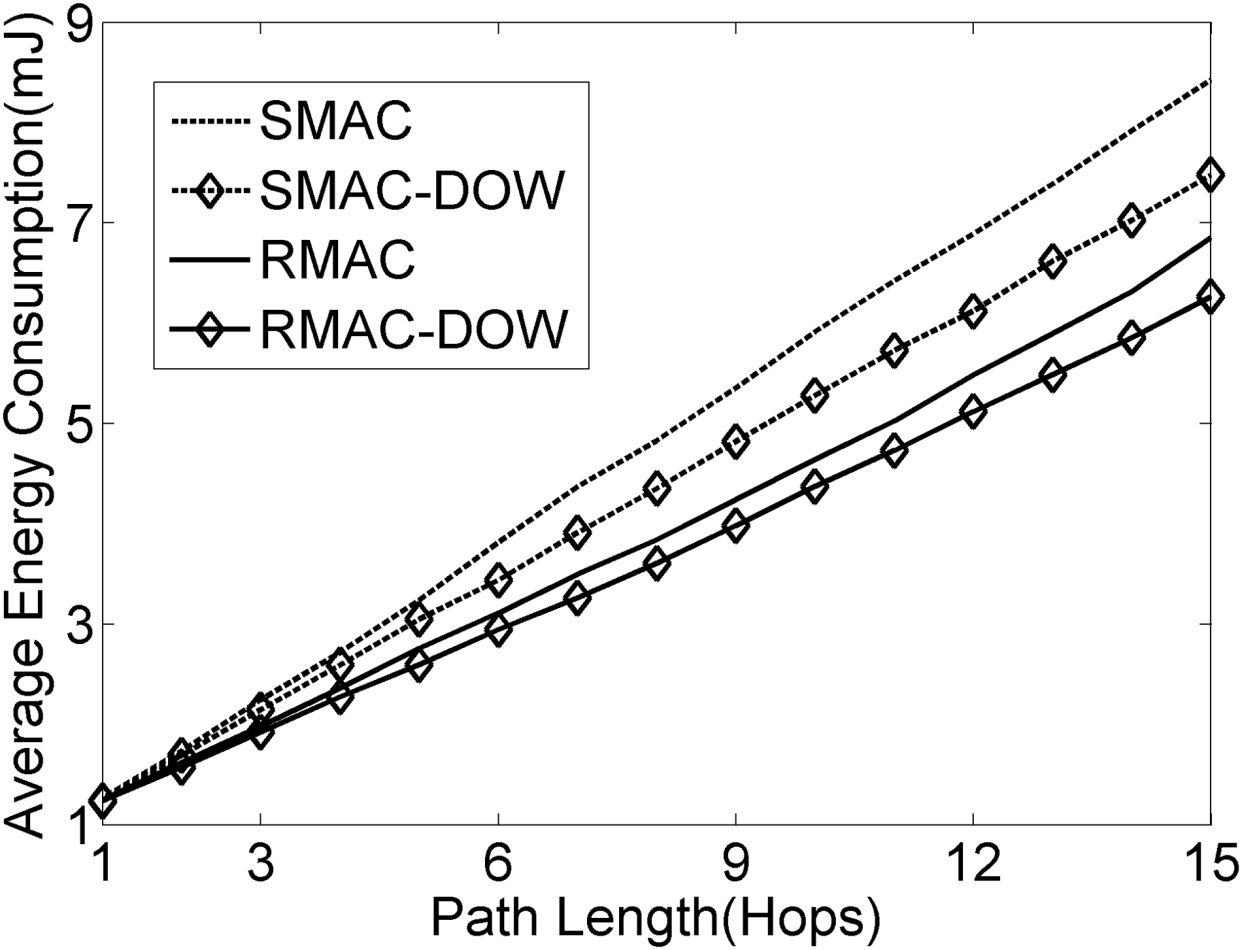
Average energy consumption versus hop count.

As shown in Figs 9 and 10, the dynamic contention window setting strategy definitely helps in further improving them when it is integrated with the existing SMAC extensions. SMAC-DOW and RMAC-DOW represent SMAC and RMAC enhanced by the dynamic optimal contention window strategy, respectively. Note that RMAC follows the similar calculation method of optimal contention window size to SMAC although there are slight differences in their frame structure. Hence, during our simulations, RMAC employs the similar calculation method of optimal contention window size to SMAC considering all frame components. It is witnessed from Fig 9 that RMAC is inferior to SMAC in the case of fewer hops. This should be ascribed to the fact that RMAC fails in effectively offering multi-hop data transmission within one single cycle in the case of the moderate traffic load. More specifically, the traffic load always tends to be relatively heavy in the regions closer to the Sink, consequently, where the collision events occur. Thus, the capability of multi-hop forwarding per operational cycle is heavily constrained. The simulations show that RMAC has to deal with medium contention on an average of 1.7 hops in the regions closer to the Sink, which is much fewer compared with the results within the extremely light traffic scenario in [19]. Additionally, it should be pointed out that the number of contending nodes within the same virtual cluster falls in the range from 2 to 7. Most virtual clusters have about 3 or 4 contending nodes. As shown in Figs 6–8, it is apparent that the dynamic contention window strategy naturally has advantages over the fixed contention window size with regards to the network performance. Therefore, this situation provides more opportunities for the dynamic contention window strategy bringing more gains to network performance. It is worthwhile to emphasize that the gain will increase at a faster rate with the increasing fluctuation in the scale of contending nodes within the same virtual cluster.

In particular, it is observed from Fig 9 that the dynamic contention window strategy offers further improvement in the end-to-end delay for RMAC than SMAC. As the hop count increases, RMAC outperforms SMAC after about nine hops, which indicates that the multi-hop data transmission mechanism in RMAC does not work well until several hops away from the Sink. The reason underneath the phenomenon is that the traffic load becomes much lighter in the regions on average several hops away from the Sink. Moreover, during the simulations, it is witnessed that there is a monotonic increase in the hop count of data transmission within one operational cycle with the increase of the distance from the Sink. From Fig 10, it is not surprised to observe that the dynamic contention window strategy effectively improves RMAC in terms of energy consumption. Additionally, the energy gains keep constantly increasing along with the increasing hop count due to the accumulation of energy savings. Based on the discussion above, it is natural to draw such conclusion: the dynamic contention window strategy is able to offer such capability of effectively improving the existing SMAC extensions, and the increasing gains will be achieved in terms of delay, throughput and energy consumption with the increase of the traffic load injected into network. Accordingly, it could reasonably be ascertained that the dynamic contention window strategy would bring more gains for the existing SMAC extensions if the routing mechanisms were taken into consideration. This is because the routing mechanisms could incur the routing information maintenance overhead. In conclusion, the dynamic contention window strategy helps in improving the effectiveness and flexibility of SMAC-like protocols by enhancing their capabilities of adaptively handling the medium contention under various traffic models and contending nodes.

### Setting strategy of contention window size

Through the simulations and curve analyses, it is revealed that there is no difference between the delay-optimization-oriented contention window sizes derived from the perspectives of single node and the whole network. As for the SMAC frame components, RTS/CTS, SYNC and EIFS sequentially follow an increasing order when it comes to the degree of their impacting the expected delivery delay, throughput and energy consumption. Compared with the traditional contention window setting strategies, the dynamic setting methods offer one better opportunity for improving the expected delivery delay, throughput and energy efficiency resulting from its inherently attempting to achieve good balance between the collision probability and idle listening duration. It is demonstrated that the ideal contention window size, aiming at optimizing the delivery delay, is dependent on the network scale, RTS/CTS, SYNC and EIFS. Therefore, one ideal setting strategy of delay-optimization-oriented SMAC contention window size requires the combination of the network scale, SYNC, RTS/CTS and EIFS. In particular, for the purpose of optimizing the key network performance, all of the aforementioned factors should be taken into consideration when one tries to develop the setting strategies of the delay-optimization contention window size.

It is discovered that the multi-hop data transmission strategies in most existing SMAC extensions, such as T-MAC and RMAC is constrained a lot due to frequent collision events in the case of bursty and dynamic traffic load, although their goal is to improve the multi-hop delay, throughput and energy consumption by trying to reserve channel along as many hops as possible. Thus, for SMAC-like protocols, the channel contention is essential for data transmission in most occasions except that the channels are already reserved in advance. As a matter of fact, the channel reservation has to suffer low success ratio for the scenarios with bursty or heavy traffic load. However, most of them follow the fixed contention window size in the canonical SMAC, instead of taking into consideration the influence of contention window size on the delivery delay. As a result, our research fills in the gaps, and is capable of providing one useful complement to these SMAC extensions. Moreover, the additional gains are confirmed in this paper through a serial of integration simulations and discussions. Overall, the dynamic contention window setting strategy definitely improves the effectiveness and flexibility of the existing SMAC extensions under various conditions of traffic models and contending nodes, resulting from the enhancement of adaptively handling the medium contention.

## Conclusions

The delivery delay is derived within the network running SMAC, from the perspectives of single node and the whole network, respectively. Through the combination of simulations and curve analyses, it is revealed that one ideal setting strategy of delay-optimization-oriented SMAC contention window size requires the combination of the network scale, SYNC, RTS/CTS and EIFS. Moreover, the dynamic setting methods offer one potentially good option for improving the expected delivery delay, throughput and energy efficiency. In order to optimize the network performance, all of the aforementioned factors should be taken into consideration when one tries to develop the setting strategies of the delay-optimization contention window. This research fills in the gaps neglected by most existing SMAC extensions. As one complementary research, the proposed setting strategy is proved to help in further improving them with respect to the end-to-end delay, throughput and energy consumption when it is integrated with the existing SMAC extensions. For energy-constraint wireless sensor networks, the energy-optimization-oriented contention window strategy is essential for improving energy efficiency and prolonging the network lifetime. In future, to further explore contention window setting methods, we will implement more research on energy-optimization-oriented contention window size and the joint-optimization of delay- and energy- oriented contention window size, although the presented results show the energy efficiency is simultaneously improved when focusing on minimizing the delivery delay.

## Supporting information

S1 File Relevant data. (RAR)

## References

[1] L Misbah, G Abdullah, A Mohammad Hossein, Ab H Siti Hafizah, A Adnan, K Muhammad Khurram, et al Distance Based and Low Energy Adaptive Clustering Protocol for Wireless Sensor Networks. PLoS ONE, 2016, 11(9):1–29. 10.1371/journal.pone.0161340 PMC5033373 27658194

[2] Sanay A, Nima Jafari N. Deployment strategies in the wireless sensor network: A comprehensive review. Computer Communications, 2016, 91–92:1–16.

[3] Asudeh A, Zaruba GV, Das SK. A general model for MAC protocol selection in wireless sensor networks. Ad Hoc Networks, 2016, 36(Part1):189–202.

[4] Byun H, Yu J. Adaptive Duty Cycle Control with Queue Management in Wireless Sensor Networks. IEEE Transactions on Mobile Computing, 2013, 12(6): 1214–1224.

[5] Hammoudeh M, Newman R. Adaptive routing in wireless sensor networks: QoS optimisation for enhanced application performance. Information Fusion, 2015, 22:3–15.

[6] M Muhammad Adeel, S Winston K.G.., W Ian. Reliability in wireless sensor networks: A survey and challenges ahead. Computer Networks, 2015, 79:166–187.

[7] Ye W, Heidemann J, Estrin D. Medium Access Control with Coordinated Adaptive Sleeping for Wireless Sensor Networks. IEEE/ACM Transactions on Networking, 2004, 12(3):493–506.

[8] Huang P, Xiao L, Soltani S, Mutka W.M., Xi N. The Evolution of MAC Protocols in Wireless Sensor Networks: A Survey. IEEE Communications Surveys & Tutorials, 2013, 15(1), 101–120.

[9] Tadayon N, Khoshroo S, Askari E, Wang H, Michel H. Power management in SMAC-based energy-harvesting wireless sensor networks using queuing analysis. Journal of Network & Computer Applications, 2013, 36(3):1008–1017.

[10] Li Y, Wen-Xian J. SMAC Protocol for Wireless Sensor Adjustment Mechanism of the Adaptive Duty Cycle. Computer Science, 2012, 39(10): 86–89.

[11] Xu L, Liang-Lun C, Shi-Liang L. Adaptive node scheduling algorithm for target tracking in wireless sensor networks. Journal on Communications, 2015,36(4):1–7.

[12] Siddiqui S, Ghani S. ES-MAC: Energy Efficient Sensor-MAC protocol for Wireless Sensor networks. In: Proceedings of the 2013 IEEE 10th International Conference on Networking, Sensing and Control (ICNSC); 2013. p. 28–33.

[13] Messaoud D, Djamel D, Nadjib B, Abdelmadjid B. Synchronous contention-based MAC protocols for delay-sensitive wireless sensor networks: A review and taxonomy. Journal of Network and Computer Applications, 2014, 38: 172–184.

[14] Jun L, lin-he J, Chen H. Analysis model of SMAC performance in WSNs. Scientia Sinica (Informatics), 2010, 40(11): 1464–1472.

[15] Wang C, Chen Y, Hou Y. The analysis and improvement of SMAC protocol for Wireless sensor networks. In: Proceedings of the 2013 IEEE 9th International Conference on Mobile Ad-hoc and Sensor Networks (MSN); 2013. p. 437–441.

[16] Yuan R, Yi-ming C, Chen D, Zhao-hui J, Jun Z, Lei-yang F, et al Performance analysis and simulation verification of S-MAC for wireless sensor networks. Computers & Electrical Engineering, 2016,56:468–484.

[17] Sakya G, Sharma V. Performance analysis of SMAC protocol in wireless sensor networks using network simulator (Ns-2) Quality, reliability, security and robustness in heterogeneous networks. Springer; 2013 42–51.

[18] Chang LW, Huang YM, Lin CC. Performance analysis of S-MAC protocol. International Journal of Communication System, 2013,26:1129–1142.

[19] S. Du, A. K. Saha, D. B. Johnson. RMAC: A routing-enhanced duty-cycle MAC protocol for wireless sensor networks. In: Proceedings of INFOCOM; 2007. p. 1478–1486.

[20] Donmez M Y, Isik S, Ersoy C. Combined analysis of contention window size and duty cycle for throughput and energy optimization in wireless sensor networks. Computer Networks, 2013, 57(5): 1101–1112.

[21] Cheng L, Kui-ru W, Jin-long Z, De-xin Z, Wang L. Optimization of listening time of S-MAC for wireless sensor networks. The Journal of China Universities of Posts and Telecommunications, 2009, 16(5): 41–45.

[22] Yun-Peng L, Chang-Biao X, Lin L. Contention window adaptive MAC protocol for wireless sensor networks. Transducer and Microsystem Technologies, 2010 (1): 49–51.

[23] Demirkol I, Ersoy C. Energy and delay optimized contention for wireless sensor networks. Computer Networks, 2009, 53(12): 2106–2119.

[24] Varga A. The OMNeT++ discrete event simulation system. Version 4.3. User Manual. URL: http://www.OMNeTpp.org, 2015.

